# Transition from Oxycodone to Buprenorphine/Naloxone in a Hospitalized Patient with Sickle Cell Disease: A Case Report

**DOI:** 10.1007/s11606-021-07295-2

**Published:** 2022-01-06

**Authors:** Sarah Leyde, Leslie Suen, Lisa Pratt, Triveni DeFries

**Affiliations:** 1 Division of General Internal Medicine, Department of Medicine, Harborview Medical Center, University of Washington, Seattle, WA USA; 2 National Clinician Scholar Program, Philip R. Lee Institute of Health Policy Studies, University of California, San Francisco, San Francisco, CA USA; 3 San Francisco Veteran Affairs Medical Center, San Francisco, CA USA; 4 San Francisco Department of Public Health, Jail Health Services, San Francisco, CA USA; 5 Division of General Internal Medicine, Department of Medicine, San Francisco General Hospital and Trauma Center, University of California, San Francisco, 1001 Potrero Ave, Ward 13, San Francisco, CA USA

**Keywords:** sickle cell disease, buprenorphine, chronic pain, vaso-occlusive episode

## Abstract

Buprenorphine is increasingly used to treat pain in patients with sickle cell disease but optimal timing and approach for transitioning patients from full agonist opioids to buprenorphine is unknown. We present the case of a 22-year-old woman with sickle cell disease and acute on chronic pain who transitioned from high-dose oxycodone to buprenorphine/naloxone during a hospital stay for vaso-occlusive episode. Utilizing a microdosing approach to minimize pain and withdrawal, buprenorphine/naloxone was gradually uptitrated while she received full agonist opioids. During the transition, she experienced some withdrawal in the setting of swallowed buprenorphine/naloxone tablets, which were intended to be dosed sublingually. Nevertheless, the transition was tolerable to the patient and her pain and function significantly improved with buprenorphine treatment. This case also highlights the challenges and unique considerations that arise when providing care for the hospitalized patient who is also incarcerated.

## BACKGROUND/CONTEXT

Many patients with sickle cell disease experience chronic pain with acute pain flares due to vaso-occlusive episodes. While full agonist opioids are first-line treatment for severe, acute pain, there is little evidence to suggest that escalating doses of full agonist opioids provide benefit for chronic pain.^1^ Buprenorphine, a partial mu opioid receptor agonist and kappa receptor antagonist, is increasingly used to treat chronic pain in sickle cell disease and previous research suggests safety, feasibility, and reduced acute care utilization.^2,3^ Yet, optimal timing and approach for transitioning patients with sickle cell disease from full agonist opioids to buprenorphine is unknown. Microdosing approaches are different from traditional buprenorphine initiation approaches. During buprenorphine microdosing, small amounts of buprenorphine are introduced to facilitate a “gentle loading of the high binding affinity and long half-life buprenorphine without significant displacement of full agonist opioid,”^4^ lowering risk of acute precipitated withdrawal. These buprenorphine initiation approaches are increasingly used to start patients on buprenorphine while minimizing opioid withdrawal.^3^

## CASE PRESENTATION

The patient is a 22-year-old woman with sickle cell disease and chronic pain on oxycodone 160 mg every 6 h (960 morphine milligram equivalents) who was admitted to the hospital from jail for a vaso-occlusive episode. She was diagnosed with sickle cell disease in childhood and experienced numerous complications including vaso-occlusive episodes, acute chest syndrome, and chronic pain. As an adolescent, she was started on opioids to treat chronic pain. Over years, escalating doses of opioids were not effective in treating pain and caused side effects such as severe constipation and sedation, which impaired her function and prevented her from doing things she enjoyed like writing song lyrics. Attempts to taper opioids were interrupted by frequent hospitalizations for vaso-occlusive episodes, where she was often discharged on higher doses of opioids. She did not readily engage with a multidisciplinary sickle cell disease team and was not receiving evidence-based treatments like hydroxyurea. At the age of 20, she was incarcerated, and Jail Health clinicians assumed opioid prescribing.

During this hospitalization, she was admitted to the hospital medicine service for management of acute on chronic pain with intravenous (IV) hydromorphone 4 mg, as needed, every 6 h, in addition to her outpatient dose of oxycodone 160 mg every 6 h. The patient inquired whether buprenorphine could be helpful for her chronic pain. The inpatient addiction consultation service was consulted to assist with a possible microdose buprenorphine transition for the patient on hospital day 2.

The patient described desire to discontinue opioids for lack of efficacy in treating her pain and because she perceived that she was treated differently for being on high doses of oxycodone. Given a family history of substance use disorders, she also worried that she could develop an opioid use disorder. In jail, she observed others experience rapid relief from opioid withdrawal with buprenorphine so she wondered if she could use this medication to taper off oxycodone.

Clinicians were concerned that transitioning to buprenorphine during the hospitalization could precipitate worsening pain because she was experiencing acute pain during a vaso-occlusive episode. Several options were discussed including (1) waiting until pain levels returned to baseline and transitioning to buprenorphine/naloxone in jail, or (2) transitioning to buprenorphine during her inpatient stay, utilizing a microdose approach to minimize withdrawal symptoms.^5^ The Jail Health team lacked the clinical resources to monitor a microdose transition in jail. The patient strongly preferred transition in the hospital setting with closer monitoring and access to medications to rapidly treat symptoms of withdrawal if needed.

**Figure 1:**
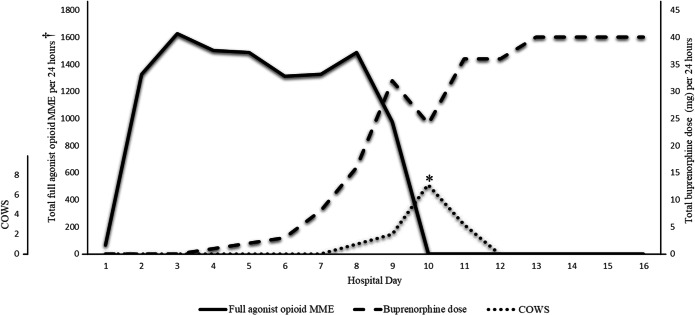
Buprenorphine microdose transition in a patient with sickle cell disease hospitalized for a vaso-occlusive episode. Max COWS score of 7 occurred on hospital day 10 with PO administration (instead of usual sublingual administration) (asterisk symbol). Full agonist opioids included IV hydromorphone and PO oxycodone (dagger symbol). Abbreviations: COWS, Clinical Opioid Withdrawal Scale; MME, morphine milligram equivalents.

On hospital day 5, while she was still experiencing acute on chronic pain, she started buprenorphine with a gradual microdose approach, starting with a 20 mcg/h buprenorphine patch while full agonist opioids were continued. Over the next 5 days, buprenorphine was slowly increased, and oxycodone was tapered (Figure 1). Her clinicians discussed each change in dosing with the patient beforehand and utilized shared decision-making for all dose adjustments (Table 1). Her maximum Clinical Opioid Withdrawal Scale score was 7 on hospital day 10, which occurred in the setting of swallowing buprenorphine/naloxone tablets instead of taking them sublingually. She remained hospitalized for several days to treat her symptoms and gradually titrated the buprenorphine/naloxone to a dose of 40/10 mg/day (in four divided doses). Despite mild to moderate withdrawal symptoms, the patient tolerated the transition well with the help of adjunctive medications like loperamide, clonidine, diphenhydramine, acetaminophen, ibuprofen, and trazodone. She also utilized mind-body relaxation strategies, music, and coloring books which helped her tolerate her withdrawal symptoms. She gave verbal and written consent to share this case report and her written reflection (Figure 1).

**Table 1:** Buprenorphine Microdose Transition in a Patient with Sickle Cell Disease Hospitalized for a Vaso-occlusive Episode

Hospital day	Full agonist opioid morphine milligram equivalents (per 24-h period)	Full agonist opioids administered (per 24-h period)	Buprenorphine administered (per 24-h period)	Maximum pain score (0–10 scale)	Maximum Clinical Opioid Withdrawal Scale (COWS) score
1 Admitted to hospital	64*	• Hydromorphone IV 4 mg × 4	--	9	0
2 Addiction specialists consulted	1328	• Oxycodone 160 × 5 • Hydromorphone IV 4 mg × 8	--	9	0
3	1626	• Oxycodone 160 × 6, 60 × 1 • Hydromorphone IV 4 mg × 6	--	10	0
4	1504	• Oxycodone 160 mg q6 hours × 6 • IV hydromorphone 4 mg q6 hours PRN × 4	--	10	0
5	1488	• Oxycodone 160 mg q6 hours × 6 • Hydromorphone IV 4 mg q6 hours PRN × 3	20 mcg/h buprenorphine transdermal patch (equivalent to ~1 mg SL buprenorphine daily)	10	0
6	1312	• Oxycodone 160 mg q6 hours × 5 • Hydromorphone IV 4 mg q6 hours PRN × 3 • Hydromorphone 16 mg PO q3 hours PRN × 1	Added 2^nd^ 20 mcg/h transdermal buprenorphine patch (two patches equivalent to ~2 mg SL buprenorphine daily)	9	0
7	1328	• Oxycodone 160 mg q6 hours ×5 • Hydromorphone 4 mg IV q6 hours PRN × 4 • Hydromorphone 16 mg PO ×1	Added 3^rd^ 20 mcg/h transdermal buprenorphine patch (three patches equivalent to ~3 mg SL buprenorphine daily)	10	0
8	1488	• Oxycodone 160 mg q6 hours ×6 • Hydromorphone 4 mg IV q6 hours PRN × 3	Three patches discontinued Buprenorphine/naloxone 2/0.5 mg SL q2 hours × 4	9	1
9	976	• Oxycodone 160 mg q4 hours ×4 • Hydromorphone 4 mg IV q6 hours PRN ×1	Buprenorphine/naloxone 8/2 mg SL × 1 Buprenorphine/naloxone 2/0.5 mg SL q2 hours × 4	10	2
10	0	--	Buprenorphine/naloxone 4/1 mg SL† q4 hours ×6 Buprenorphine/naloxone 8/2 mg SL* ×1	9	7
11	0	--	Buprenorphine/naloxone 8/2 mg SL† q 8 hours ×3	10	3
12	0	--	Buprenorphine/naloxone 8/2, 8/2, 8/2, 12/3 mg (q6 hour)	8	0
13	0	--	Buprenorphine/naloxone 8/2, 8/2, 8/2, 12/3 mg	8	Stopped checking COWS
14	0	--	Buprenorphine/naloxone 12/3, 8/2, 8/2, 12/3 mg	8	--
15	0	--	Buprenorphine/naloxone 12/3, 8/2, 8/2, 12/3 mg	6	--
16 Discharged mid-day	0	--	Buprenorphine/naloxone 12/3, 8/2 mg	5	--

*She received a total of 64 MME of full agonist opioids while hospitalized on hospital day 1 but may have received other opioids on that day in jail prior to being admitted

†In the setting of taking buprenorphine orally, not sublingually. On this day, she was also treated with clonidine 0.2 mg q6 hours PRN opioid withdrawal, loperamide, and mind-body relaxation strategies. Changed from buprenorphine tablets to films to avoid confusion about oral versus sublingual administration

On hospital day 16, the patient was discharged back to jail. She was briefly readmitted to the hospital with acute worsening of pain after a missed dose of buprenorphine in the jail. During this subsequent hospitalization, buprenorphine/naloxone was increased to 48 mg/12 mg daily in four divided doses. After close coordination with Jail Health Services to ensure timely dosing, she has experienced marked improvement in pain levels, reporting 0/10 pain level at 4 months after starting buprenorphine, as well as improved mood and function (Text Box 1).

**Text Box 1** Patient and provider perspectives on a buprenorphine microdose transition in a patient with sickle cell disease hospitalized for a vaso-occlusive episode

**Table:** 

**Patient perspective (delivered to team via a hand-written note from the patient at the end of her first hospitalization):** “During the transformation from taking oxycodone to using the buprenorphine was not very easy but also doable. With the oxy I just built up a tolerance which the meds stop effecting me pain wise and was only giving a sleepy/mood swings side effects, which in my opinion feel like it was slowing me down more than anything. With the buprenorphine I feel more energy, more clearheaded and some sort of relief. If it was up to me and I had things my way I wouldn’t be taking no medication at all. But unfortunately you just don’t get that lucky. Within the process I still did have a lot of diarrhea and chills but as the days pass it gets better. I feel that the switch was very helpful and necessary. I would recommend it for anybody.” **Jail Health MD perspective (emailed to team 4 months after initial hospital visit):** “She has had no concern of pain! None. She is relaxed, engaged, pleasant and, per her report, the first time in about 10 years that she has felt happy. She has requested to be a worker in the jail – a huge change from her first two years here where she never was out of bed other than to shower and eat. She slept most of the day before [the transition]. Please pass along that this has been an unmitigated success.”	

## DISCUSSION

We present a case of a microdose transition from high-dose oxycodone to buprenorphine in a patient hospitalized for vaso-occlusive episode of sickle cell disease. Previous case reports and series of buprenorphine treatment for pain in patients with sickle cell disease suggest safety, acceptability, and decreased healthcare utilization.^6–8^ This case is notable for several reasons:

In contrast to the traditional method of starting buprenorphine in which patients stop full agonist opioids and wait for moderate opioid withdrawal symptoms to occur, we utilized a microdose bridge to minimize withdrawal and allow for better pain control during an acute pain episode. A recently published case report describes using this approach for two adolescent patients with sickle cell disease in the outpatient setting, but our report is the first to describe this approach during an inpatient hospitalization.^9^ Complex transitions to buprenorphine can be challenging in the outpatient setting. Hospitalization presents an opportunity to carefully monitor and rapidly provide adjunctive agents to maximize comfort. Furthermore, for this patient experiencing incarceration, the hospital setting allowed more patient autonomy and shared decision-making about medical treatment than may have been available in jail. The hospitalization also provided a unique opportunity for close coordination with Jail Health, Hematology, Addiction Medicine, and Hospital Medicine teams.

This case also highlights challenges of complex buprenorphine transitions taking place in the hospital. The patient swallowed buprenorphine after being administered a cup of several oral medications including buprenorphine tablets. Buprenorphine has poor oral bioavailability due to extensive 1^st^ pass metabolism and should be administered sublingually.^7^ This ultimately led to subtherapeutic dosing, possibly lengthening the hospital stay.

This report is also unique in terms of the high dose of buprenorphine this patient required for analgesia. Although there is no consensus on optimal dosing of buprenorphine/naloxone in patients transitioned from high-dose full agonist opioids, existing literature in patients with OUD suggests that doses of at least 16 mg are associated with higher retention in treatment and decreased extra-medical opioid use. It is usual practice to adjust the dose until withdrawal and cravings are suppressed, typically requiring ≥ 16 mg in a patient with high opioid tolerance.^8^ In this case, the dose of buprenorphine needed to control pain was much higher than typical. We suspect this may be due to the patient’s very high dose of oxycodone prior to the transition leading to upregulation of mu opioid receptors. Although it is rare for patients to require more than 32 mg of buprenorphine, early pharmacodynamic research suggests that while there is a ceiling to the CNS and respiratory effects of buprenorphine, there is no definitive analgesic ceiling effect.^10^

Lastly, this report highlights the unique challenges caring for patients within the carceral system, who historically have had limited access to buprenorphine, let alone to addiction medicine specialists who can manage complex transitions onto buprenorphine.^11^ Prior to transitioning to buprenorphine, we discussed with the jail medical director the Jail Health system’s capacity to continue with this treatment at the higher doses and frequent dosing schedule required for chronic pain management. Inside the hospital, this patient, like many who are in custody, was shackled to the bed and unable to mobilize when experiencing pain. In efforts to build trust and support the patient undergoing withdrawal and pain, we found it helpful to ask officers to leave the room for privacy, provide music and art supplies for multimodal pain management, offer gestures of comfort, and facilitate communication with other specialist medical providers for sickle cell disease who were difficult to access while in jail.^12^

## CONCLUSION

Our case report suggests that microdose transition from chronic, high-dose full agonist opioids to buprenorphine in patients with sickle cell disease, even during a hospitalization for an acute vaso-occlusive episode, may be safe, effective, and feasible. More research is needed to understand the optimal transition strategies and dosing of buprenorphine among people with sickle cell disease.
